# Novel Murine Biomarkers of Radiation Exposure Using An Aptamer-Based Proteomic Technology

**DOI:** 10.3389/fphar.2021.633131

**Published:** 2021-04-26

**Authors:** Mary Sproull, Uma Shankavaram, Kevin Camphausen

**Affiliations:** Radiation Oncology Branch, National Cancer Institute, Bethesda, MD, United States

**Keywords:** biomarker, somascan, biodosimetry, radiation, medical countermeasure

## Abstract

**Purpose:** There is a need to identify new biomarkers of radiation exposure both for use in the development of biodosimetry blood diagnostics for radiation exposure and for clinical use as markers of radiation injury. In the current study, a novel high-throughput proteomics screening approach was used to identify proteomic markers of radiation exposure in the plasma of total body irradiated mice. A subset panel of significantly altered proteins was selected to build predictive models of radiation exposure and received radiation dose useful for population screening in a future radiological or nuclear event.

**Methods:** Female C57BL6 Mice of 8–14 weeks of age received a single total body irradiation (TBI) dose of 2, 3.5, 8 Gy or sham radiation and plasma was collected by cardiac puncture at days 1, 3, and 7 post-exposure. Plasma was then screened using the aptamer-based SOMAscan proteomic assay technology, for changes in expression of 1,310 protein analytes. A subset panel of protein biomarkers which demonstrated significant changes (*p* < 0.05) in expression following radiation exposure were used to build predictive models of radiation exposure and radiation dose.

**Results:** Detectable values were obtained for all 1,310 proteins included in the SOMAscan assay. For the Control vs. Radiation model, the top predictive proteins were immunoglobulin heavy constant mu (IGHM), mitogen-activated protein kinase 14 (MAPK14), ectodysplasin A2 receptor (EDA2R) and solute carrier family 25 member 18 (SLC25A18). For the Control vs. Dose model, the top predictive proteins were cyclin dependent kinase 2/cyclin A2 (CDK2. CCNA2), E-selectin (SELE), BCL2 associated agonist of cell death (BAD) and SLC25A18. Following model validation with a training set of samples, both models tested with a new sample cohort had overall predictive accuracies of 85% and 73% for the Control vs. Radiation and Control vs. Dose models respectively.

**Conclusion:** The SOMAscan proteomics platform is a useful screening tool to evaluate changes in biomarker expression. In our study we were able to identify a novel panel of radiation responsive proteins useful for predicting whether an animal had received a radiation exposure and to what dose they had received. Such diagnostic tools are needed for future medical management of radiation exposures.

## Introduction

Mass casualty medical management of potential radiological or nuclear events primarily require diagnostics to effectively identify individuals who have received a radiation exposure. Many promising approaches are currently under development including point-of-care and high-throughput off-site approaches (Garty et al., 2016; Balog et al., 2020; Jacobs et al., 2020). These diagnostics are based on physiological biomarkers of radiation injury found primarily in the blood and include a wide array of molecules at the genomic, proteomic, metabolomic, and transcriptomic level. In addition, some methodologies for characterizing radiation exposure utilize cytogenetic markers, lymphocyte depletion kinetics and electron paramagnetic resonance (EPR). Cumulatively, this variety of biomarker classes represent the complex physiologic interaction of biological mechanisms involved in ionizing radiation injury (Sproull and Camphausen, 2016). At the proteomic level several key biomarkers of radiation exposure have been established in mammalian models of radiation exposure and include Flt3 ligand (FL), a marker of hematopoietic stem cell recovery, acute phase response proteins c-reactive protein (CRP) and serum amyloid A (SAA) and other markers such as salivary alpha amylase (AMY1) and monocyte chemotactic protein 1 (MCP1) (Ossetrova et al., 2011; Sproull et al., 2017; Balog et al., 2019).

Characterization of proteomic biomarkers of radiation exposure and novel proteomic biomarkers of other disease states have previously been done using singleplex ELISA assay or using multiplex immunoassay approaches including reverse phase protein arrays (RPPAs), bead-based assays or electrochemiluminescent-antibody based technologies (Sproull et al., 2013; Boellner and Becker, 2015; Sproull et al., 2015; Himburg et al., 2016; Blakely et al., 2018; Kuang et al., 2018). Multiplex approaches have clear benefits in exploratory studies for biomarker discovery in terms of cost and efficiency as they maximize target screening using less sample volume. To date, the best of these various multiplex platforms could offer was target screening at the level of a few hundred proteomic targets. In the current study, we sought to take advantage of emerging high-throughput technologies which examine changes in the mammalian proteome through high level multiplex approaches. With access to a larger array of protein targets, changes in the mammalian proteome due to radiation injury can be better characterized. Using the innovative aptamer-based SOMA-scan proteomic assay technology, plasma from C57BL6 mice was screened for changes in expression of 1,310 protein analytes following a total body radiation exposure of 2, 3.5 or 8 Gy. A subset panel of proteins which demonstrated significant changes in expression following radiation exposure was selected to build predictive models of radiation exposure. In mass casualty medical management of events involving radiation exposure, screening to identify those individuals who have received a radiation exposure is a key element (Sullivan et al., 2013). Yet, different predictive diagnostics of radiation exposure may be needed at different levels of triage. To address this need, we created two predictive models of radiation exposure. Firstly, a “Control vs. Radiation” model was developed to predict whether an individual had received a radiation exposure and needed further triage or had not received a radiation exposure and could be sent home. We also developed a “Control vs. Dose” model to predict how great a radiation dose an individual had received which is useful for guiding subsequent medical management decisions.

For this study, model building and validation were completed with two separate sample sets to independently test the strength of the respective models. This study identifies both novel proteomic biomarkers of radiation exposure and two useful predictive models of radiation exposure using the Somalogic SOMAscan platform.

## Methods

### Animal Model

For the murine model used in this study, 8–14 week old female C57BL6 mice received a single total body irradiation (TBI) dose of 2, 3.5, 8 Gy or sham radiation. All mice receiving TBI were confined using a standard pie jig preventing movement. All animal studies were conducted in accordance with the principles and procedures outlined in the NIH Guide for the Care and Use of Animals and procedures were approved by the NIH Lab Animal Safety Program under an approved protocol. Plasma was collected by cardiac puncture using a heparinized syringe at days 1, 3, and 7 post-irradiation in Lithium Heparin blood collection tubes (BD Biosciences). Mice received 2.5–5.5% Isoflurane anesthesia during cardiac puncture for blood collection. Plasma was spun at 10,000 RCF for 10 min at 4°C and all samples were stored at −80°C.

### Dosimetry

For the murine *in vivo* model utilized in this study, a Pantak X-ray source was used at a dose rate of 2.28 Gy/min. Dose rate was calibrated based upon the procedures described in American Association of Physicist in Medicine (AAPM) Task Group Report 61 (TG-61) with regard to the following conditions: X-ray tube potential was 300 kV, half value layer (HVL) is 0.8 mm Copper (Cu), source to surface distance (SSD) was 50 cm. Dose rate was measured at 2 cm depth in solid water phantom using a PTW model: N23342 ion chamber and Inovision, model 35040 electrometer.

### SomaLogic SOMAscan Assay

Approximately 150 ul of plasma per sample was used for the Somalogic SOMAscan Assay which uses a novel protein-capture aptamer-based technology (Rohloff et al., 2014). For this study the SOMAscan HTS Assay 1.3 K was used and processed through the Center for Human Immunology at the National Institutes of Health. The assay included measurement of 1,310 protein analytes.

### Statistical Analysis

In brief, data was received in the form of Relative Fluorescent Units (RFU) for each of the 1,310 proteins in the SOMAscan assay after normalizing for intraplate and interplate variation. These RFU scores for each protein were log2 and z-score transformed. Statistical data analysis was performed using R (Team, 2015). In this study, we investigated the effect of feature selection and prediction algorithm on the performance of prediction method thoroughly. We considered the following feature selection and prediction methods implemented sequentially: differential expression analysis, random forest, regularized regression analysis, and linear discriminant analysis (Supplementary Figure S1). For these methods, we studied the effects of feature selection and the number of features on prediction.

*Differential expression analysis:* To remove invariant data from the analysis, we first performed *t*-test or ANOVA analysis depending on whether there two groups (Control vs. RT) or multiple groups (Control vs. Dose) respectively. Significance test with (*p* ≤ 0.05) were selected for further analysis.

*Feature selection:* Random Forest (RF) is a classification algorithm using sets of random decision trees which are generated by a bootstrap sampling for decision and voting (Breiman, 2001; Banfield et al., 2007). We implemented Boruta algorithm which is a wrapper built around random forest. Boruta is a feature selection algorithm and feature ranking based on the RF algorithm. Boruta’s benefits are to decide the significance of a variable and to assist the statistical selection of important variables. Besides, we can manage the strictness of the algorithm by adjusting the *p*-value that defaults to 0.01. This method allowed us to capture all the important and interesting features with respect to the outcome variable (either RT model or Dose model).

### Elastic-Net Analysis

It is evident that good classification and prediction requires good predictors. Elastic-net regularization uses ridge and LASSO penalties simultaneously to take advantages of both regularization methods (Zou and Hastie, 2005). Elastic-net provides shrinkage and automatic variable selection. Since Elastic-net feature selection is the result of random permutations, we tend to get slightly different set of features with each iteration. Since our main goal is to find stable set of features for wider application, we implemented 20 iterations of elastic-net computations resulting in 20 independent models. We then ranked the features by how often each feature is present in maximum number of models and selected top ranked four features.

### Linear Discriminant Analysis

Linear discriminant analysis (LDA) is used to find linear combinations of features which characterize or discriminate two or more classes. LDA is simple and fast. LDA was used for the purpose of final feature selection and classification. A permutation test evaluated whether the specific classification of the individuals between groups is significantly better than random classification in any two arbitrary groups (Bylesjö et al., 2006). Finally, we performed model performance evaluation with the new data for prediction accuracy. The significance of each model and importance of each feature in the model is further tested by multivariate and univariate anova tests for both training and testing models. The results were shown as heatmap and PCA plots.

## Results

### Multivariate Model Generation

In the current study, we sought to take advantage of an emerging technology, the Somalogic SOMAscan assay, to identify novel biomarkers of radiation exposure using a multiplex-analysis approach and use these findings to build radiation exposure and dose prediction models. The radiation exposure model (Control vs. RT) was designed to differentiate only between exposed and unexposed animals with the exposed animals receiving a TBI dose between 2 and 8 Gy. The dose prediction model (Control vs. Dose) was designed to both differentiate between exposed and unexposed and between the exposed by dose (2, 3.5, and 8 Gy). To this end, values for all 1,310 SOMAmer targets were obtained for each control and radiation treated sample. SOMAmer targets which demonstrated significant changes in expression following radiation exposure were selected using an ANOVA test (*p* < 0.05) and then filtered by rank using a Random Forest analysis. From this subset of proteins, the top four ranked proteins were selected for each model. For the Control vs. Radiation (RT) model, the top predictive proteins were immunoglobulin heavy constant mu (IGHM), mitogen-activated protein kinase 14 (MAPK14), ectodysplasin A2 receptor (EDA2R) and solute carrier family 25 member 18 (SLC25A18). For the Control vs. Dose model, the top predictive proteins were cyclin dependent kinase 2/cyclin A2 (CDK2. CCNA2), E-selectin (SELE), BCL2 associated agonist of cell death (BAD) and SLC25A18. For each model, a training set of samples was used to generate the model and determine its predictive accuracy and a subsequent set of test samples was later collected and used to validate each model.

### Multivariate Control vs. RT Prediction Model

**FIGURE 1:**
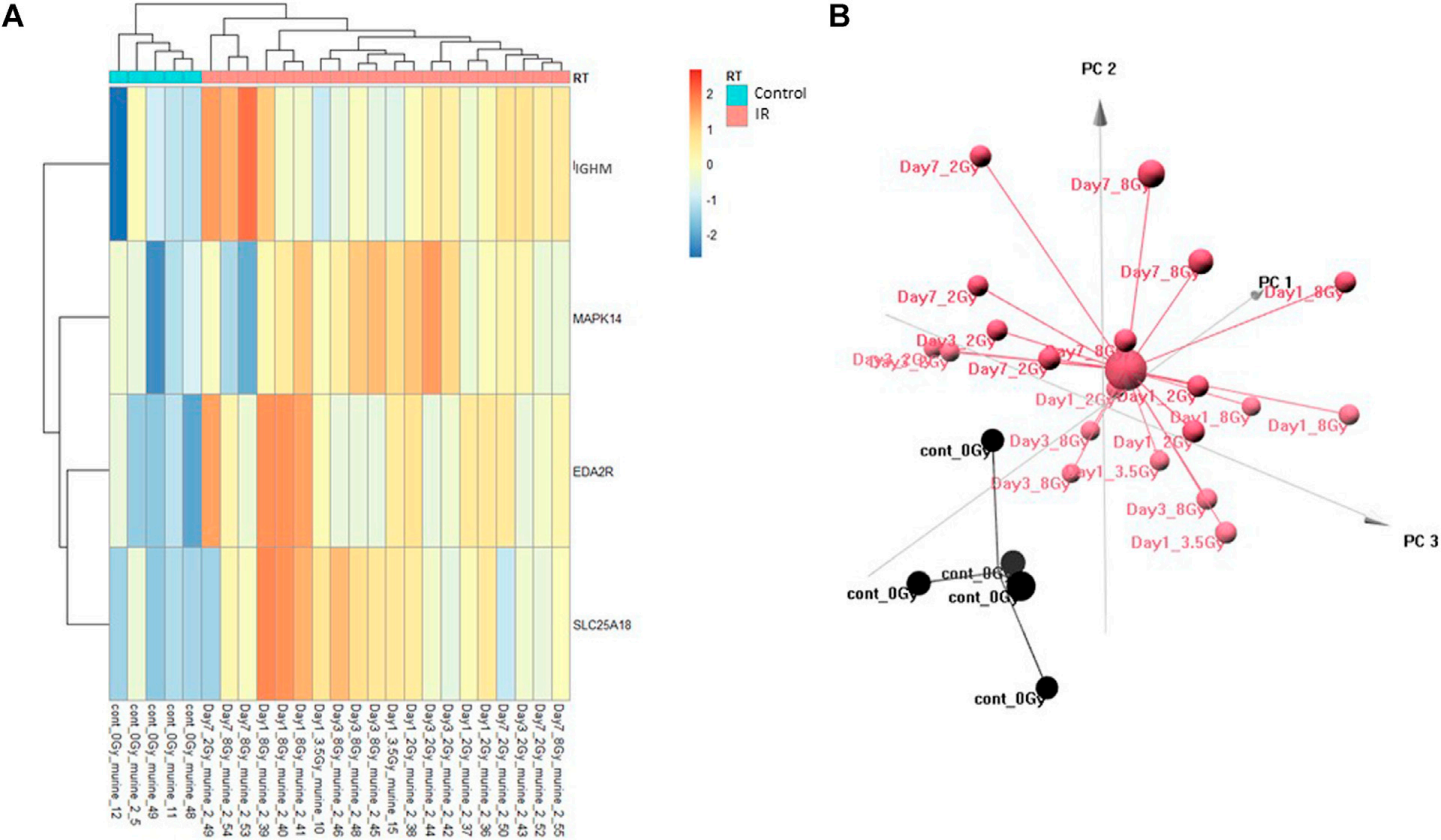
Control vs. RT Training Model. Analysis of proteomic expression within the sample cohort used for model building of the Control vs. RT Training model using top selected proteins IGHM, MAPK14, EDA2R and SL25A18. Findings are represented by **(A)** heatmap and **(B)** PCA.

**FIGURE 2:**
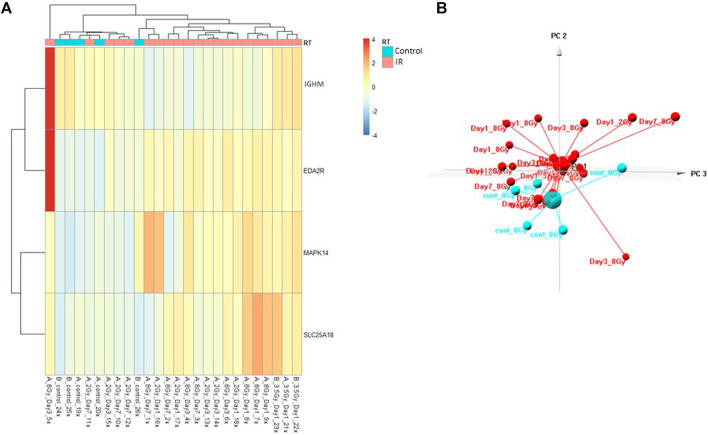
Control vs. RT Test Model. Analysis of proteomic expression within the sample cohort used for testing of the Control vs. RT Test model using top selected proteins IGHM, MAPK14, EDA2R, and SKC25A18. Findings are represented by **(A)** heatmap and **(B)** PCA.

**FIGURE 3:**
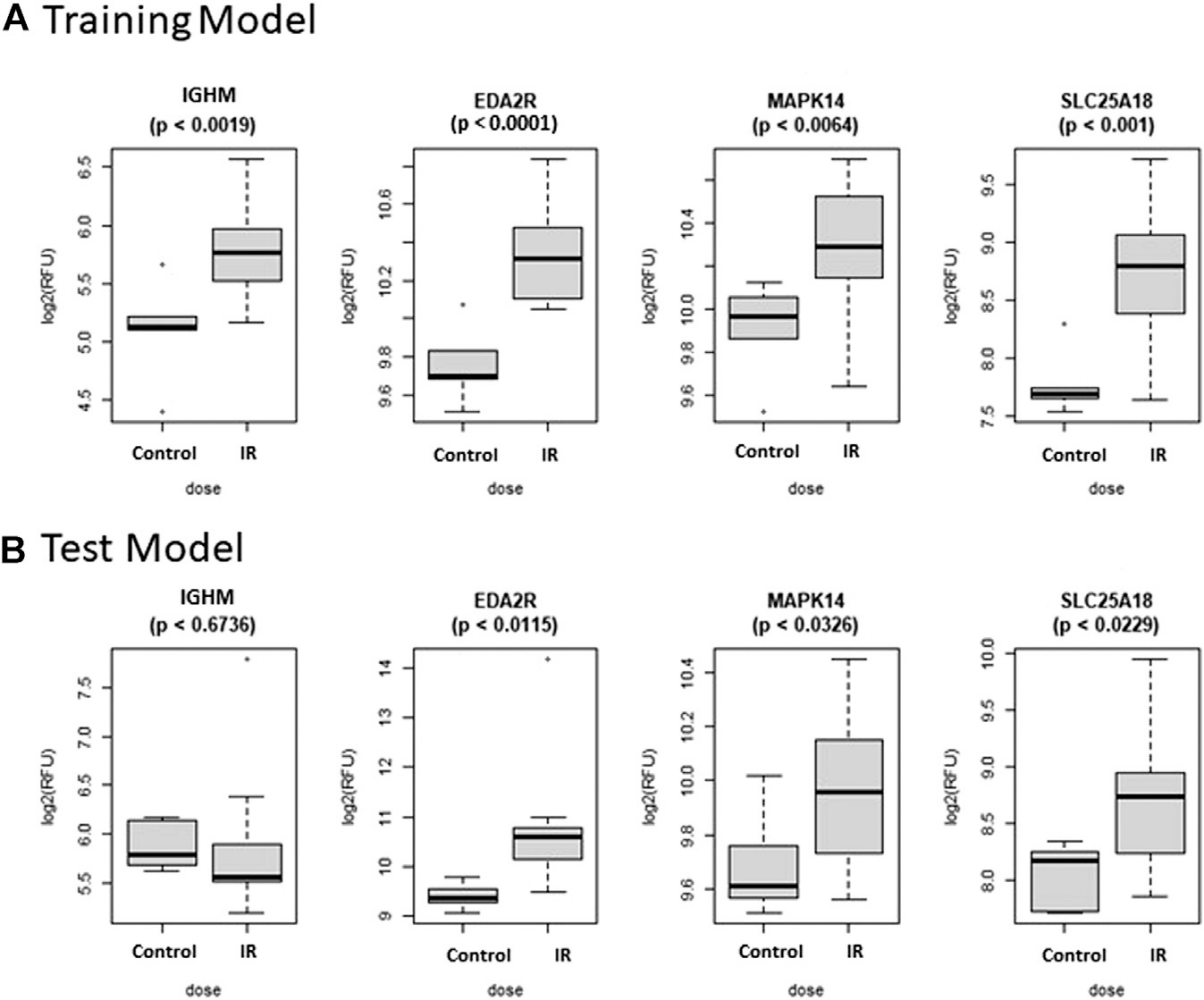
Comparison of Selected Proteins in Control vs. RT Model. Individual biomarker expression profiles for each Control vs. RT Model protein are shown for the sample sets used for both the **(A)** Training and **(B)** Test models.

The Control vs. RT prediction model was structured using SOMAmer data for IGHM, MAPK14, EDA2R, and SLC25A18. Samples included un-irradiated controls and samples from TBI C57BL6 mice receiving either 2, 3.5, or 8 Gy collected at days 1, 3 or 7 post-exposure. Samples were pooled into control and irradiated (RT) groups. Heatmap clustering of the training set of samples showed good congruency for the Control vs. RT samples (Figure 1A). Principle component analysis (PCA) of the sample grouping also showed good separation of the Control vs. RT samples for the training model (Figure 1B). Analysis of the new test set of samples used to validate the model showed less precise clustering of the Control vs. RT samples as compared to the training set. As shown in Figure 2A heatmap clustering of Control vs. RT samples was less congruent with some overlap between control and irradiated samples. Similarly, in Figure 2B, sample clustering using PCA showed less separation between the control and irradiated groups as compared to the training sample set. Illustration of the individual expression patterns for each of the proteins used in the model are shown for both the training model samples and test model samples in Figure 3. Significant changes in the expression patterns for IGHM, EDA2R, MAPK14, and SLC25A18 was seen in the training model samples as measured by Students t-test (*p* < 0.01), but only for EDA2R, MAPK14, and SLC25A18 in the test model samples (*p* < 0.05) as shown in Figures 3A,B respectively. Other than the difference in IGHM expression between the training and test model samples, all the proteins for this model showed similar trends in expression between the two sample sets.

### Multivariate Control vs. Dose Prediction Model

**FIGURE 4:**
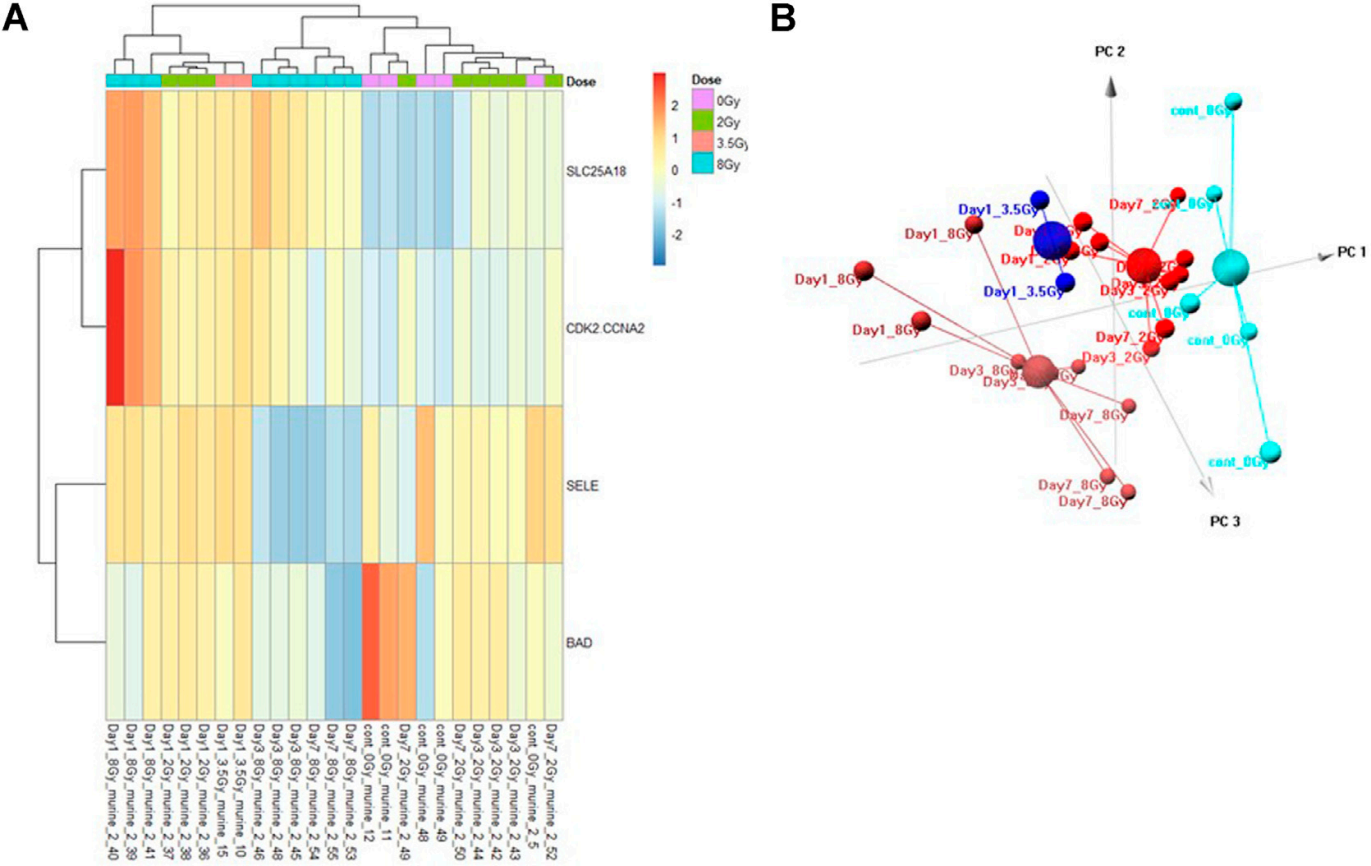
Control vs. Dose Training Model. Analysis of proteomic expression within the sample cohort used for model building of the Control vs. Dose Training model using top selected proteins SL25A18, CDK2/CCNA2, SELE, and BAD. Findings are represented by **(A)** heatmap and **(B)** PCA.

**FIGURE 5:**
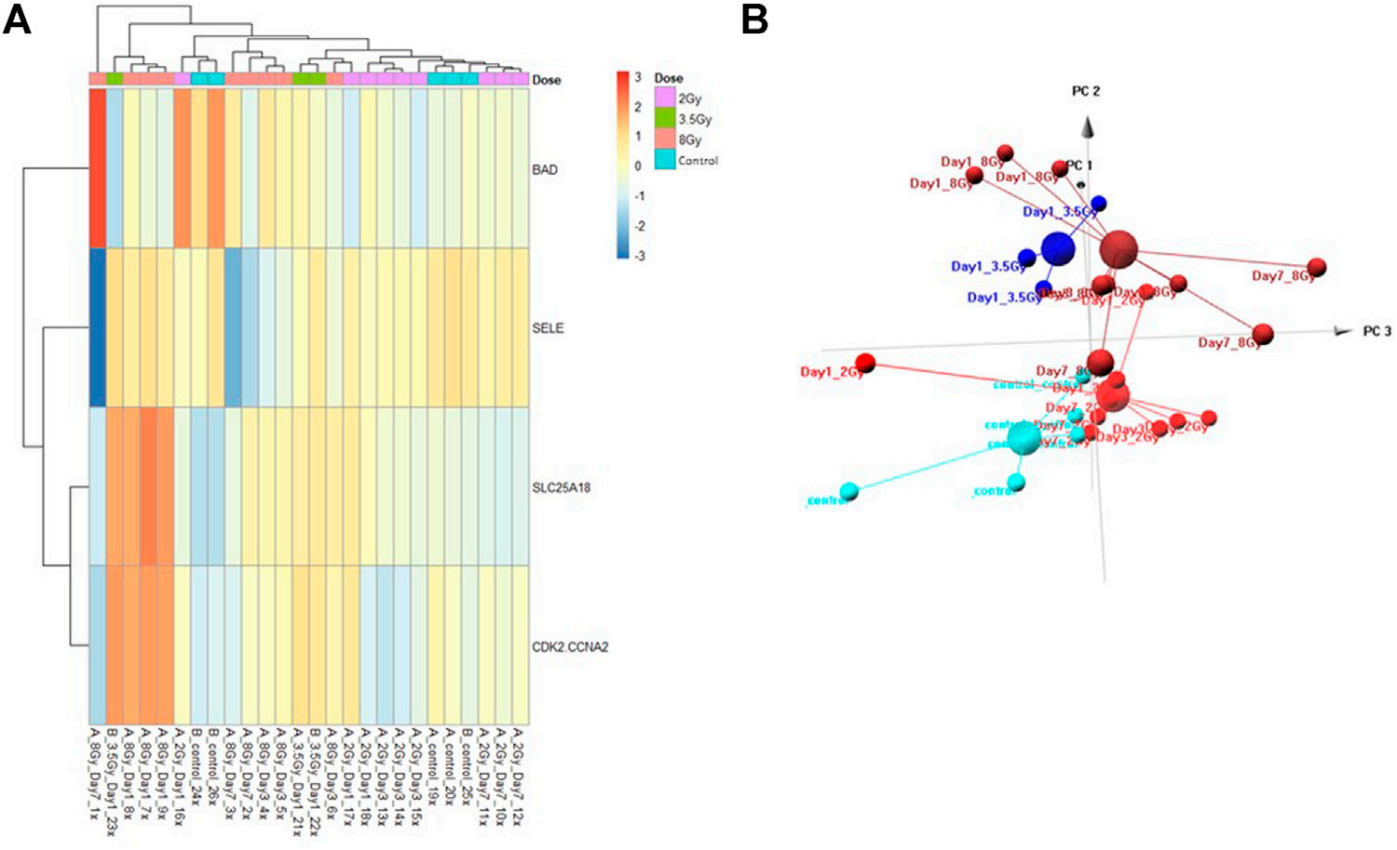
Control vs. Dose Test Model. Analysis of proteomic expression within the sample cohort used for testing of the Control vs. Dose Test model using top selected proteins SL25A18, CDK2/CCNA2, SELE, and BAD. Findings are represented by **(A)** heatmap and **(B)** PCA.

**FIGURE 6:**
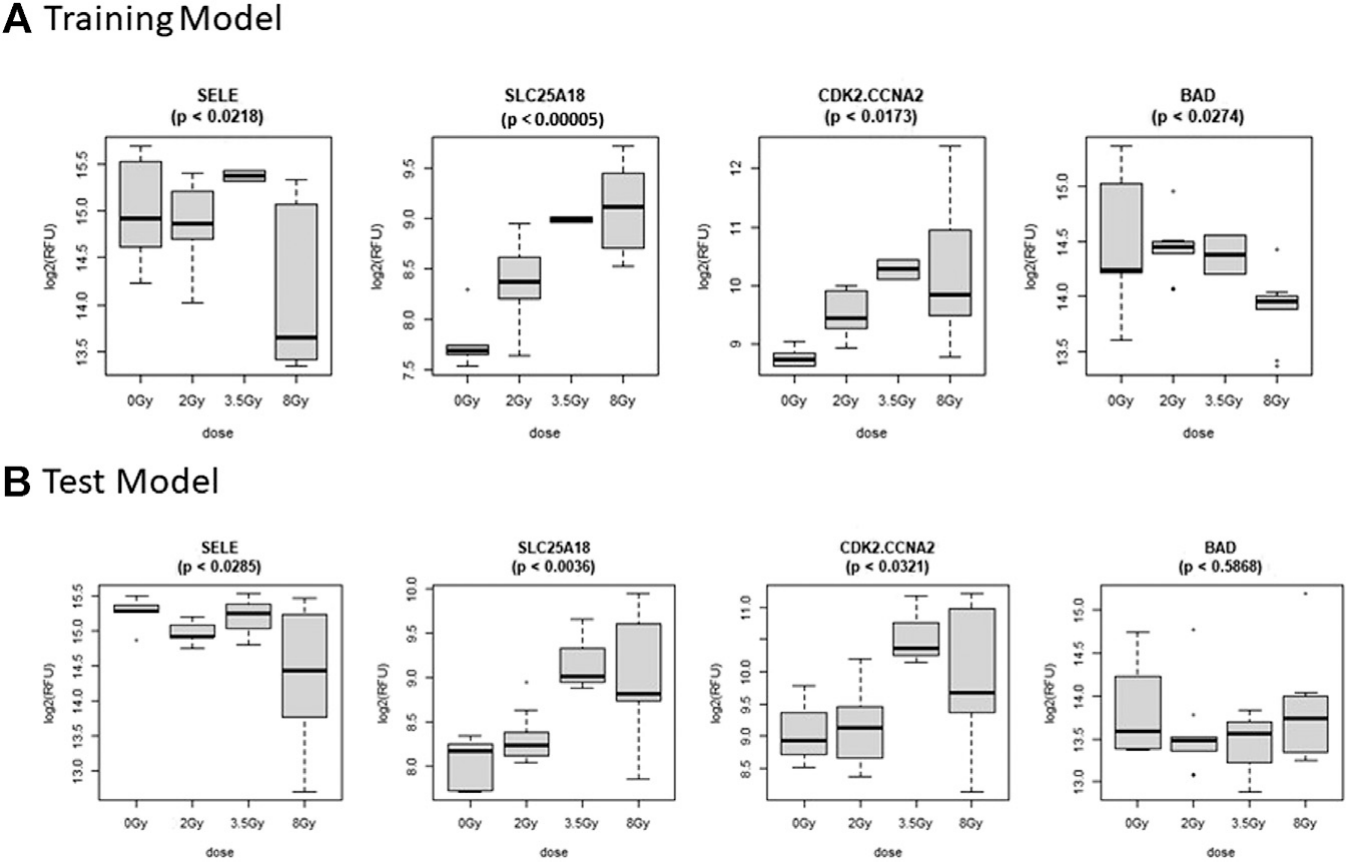
Comparison of Selected Proteins in Control vs. Dose Model. Individual biomarker expression profiles for each Control vs. Dose Model protein are shown for the sample sets used for both the **(A)** Training and **(B)** Test models.

The Control vs. Dose prediction model was structured using SOMAmer data for CDK2/CCNA2, SELE, BAD and SLC25A18. Samples included un-irradiated controls and samples from TBI C57BL6 mice receiving either 2, 3.5 or 8 Gy collected at days 1, 3 or seven post-exposure. Sample groups were determined by each dose. Heatmap clustering of the training set of samples was generally consistent yet there was some overlap between the samples as shown in Figure 4A. Similarly, PCA showed reasonable clustering within groups but some overlap between exposure groups (Figure 4B). Analysis of the new test set of samples used to validate the model showed a similar level of overlap between groups with the tightest clustering for the 8 Gy Day 1 samples (Figure 5A). PCA additionally showed some overlap between sample groups (Figure 5B). Illustration of the individual expression patterns for each of the proteins used in the model are shown for both the training model samples and test model samples in Figure 6. Significant changes in the expression patterns for SELE, SLC25A18, CDK2/CCNA2, and BAD was seen in the training model samples by Students t-test (*p* < 0.05), but only for SELE, SLC25A18 and CDK2/CCNA2 in the test model samples as shown in Figures 6A,B respectively. Comparison of respective expression trends in the Control vs. Dose model showed more variation between the training and test model sample sets than was seen in the Control vs. RT model. This is to be expected as these samples, when separated by individual dose, result in a much smaller N for each group.

### Model Variance and Predictive Accuracy

Following analysis of the expression patterns of the respective proteins within the sample cohorts used to construct and test each model, we wished to test the overall strength of each model. For both the Control vs. RT and Control vs. Dose prediction models, algorithm development using a linear discriminant analysis approach was completed with consideration for dose groups but irrespective of collection time post-exposure. To test the significance of the validation data, both a multivariate (MANOVA) and univariate approach (ANOVA) were used to test whether there were significant differences between the sample groups (Table 1). Though the multivariate test demonstrated overall significance between the test groups (control vs. RT or control vs. Dose) it does not tell us for which individual protein comparisons there is a significant observed mean differences. Therefore, a series of univariate ANOVAs were performed to determine the significance of these differences.

**TABLE 1:** Model Validation Analysis of Variance. Models were validated using both multivariate analysis of variance (MANOVA) and univariate analysis of variance (ANOVA) for both the Control vs. RT and Control vs. Dose training and test models. *p*-values represent the Pr (>F), the *p*-value of the given effect and F statistic.

Analysis of variance model validation					
Control vs. RT training model		*p* value	Control vs. Dose training model		*p* value
Multivariate analysis		3.47*E* − 06	Multivariate analysis		5.30*E* − 08
Univariate analysis			Univariate analysis		
	IGHM	0.002		SELE	0.022
	EDA2R	1.479*E* − 04		SLC25A18	4.81*E* − 05
	MAPK14	0.006		CDK.CCNA2	0.017
	SLC25A18	0.001		BAD	0.027
Control vs. RT test model			Control vs. Dose test model		
Multivariate analysis		0.008	Multivariate analysis		8.76*E* − 07
Univariate analysis			Univariate analysis		
	IGHM	0.674		SELE	0.028
	EDA2R	0.011		SLC25A18	3.60*E* − 03
	MAPK14	0.033		CDK.CCNA2	0.032
	SLC25A18	0.023		BAD	0.587

As shown in Table 1, for the Control vs. RT training model, significance was seen at both the multivariate (*p* < 0.001) and univariate level for all proteins (*p* < 0.01–0.001). The Control vs. RT test model was similarly significant at the multivariate level (*p* < 0.01) and at the univariate level for all the individual proteins (*p* < 0.05) excepting IGHM. For the Control vs. Dose training model, significant differences were seen between test groups both at the multivariate level (*p* < 0.001) and at the univariate level for all proteins (*p* < 0.05–0.001). The Control vs. Dose test model was similarly significant at the multivariate level (*p* < 0.001) and at the univariate level for all the individual proteins (*p* < 0.05) excepting BAD.

Predictive accuracy was determined for each respective model (Table 2). The Control vs. RT model had a 100% overall predictive accuracy for the training model but only 85% for the test model. The Control vs. Dose model had a 96% overall predictive accuracy for the training model and 73% for the test model. In both models the predictive accuracy decreased when the training model was tested with fresh samples. Predictive accuracies for each individual sample set demonstrate the dose groups where the model was less successful at dose prediction with only 50% predictive accuracy for the 2 Gy group in the Control vs. Dose training model and 33% predictive accuracy for the control group in the Control vs. Dose test model. These results demonstrate the relative strength of the respective models to identify which animals have received a radiation exposure and which radiation dose they have received.

**TABLE 2:** Model Predictive Accuracy. Summary of the predictive accuracy scores for each model using a linear discriminant analysis approach. Both overall predictive accuracy scores and predictive accuracy scores by test group were generated for both Control Vs. RT and Control vs. Dose training and test models.

Predictive accuracy across models					
Control vs. RT training model			Control vs. Dose training model		
Overall predictive accuracy		100%	Overall predictive accuracy		96%
Accuracy by test group			Accuracy by test group		
	Control	100%		0 Gy	100%
	RT	100%		2 Gy	50%
				3.5 Gy	100%
				8 Gy	100%
Control vs. RT test model			Control vs. Dose test model		
Overall predictive accuracy		85%	Overall predictive accuracy		73%
Accuracy by test group			Accuracy by test group		
	Control	81%		0 Gy	33%
	RT	100%		2 Gy	89%
				3.5 Gy	89%
				8 Gy	40%

## Discussion

This study presents two novel predictive models of radiation exposure using the high throughput proteomic screening Somalogic SOMAscan platform. Using a relatively small amount (150 ul) of plasma 1,310 proteins were screened for expression changes following a total body irradiation exposure. Using this approach, two predictive models of radiation exposure were built and validated with separate test samples. Both the Control vs. RT and Control vs. Dose models had good overall predictive accuracies of 85% and 73% respectively. Though the predictive accuracies for the tested models were lower than the training models, the additional step of testing each model with independent samples further validates the strength of the respective predictive algorithms. It also demonstrates model stability relative to internal technical variables intrinsic to the Somalogic SOMAscan assay and biological variables inherent to individual animals, as the samples used for the training model and the samples used to subsequently test the model were collected more than a year apart. As we have demonstrated previously, factors which affect successful application of a multivariate dose prediction algorithm include variation in technical and biological variables (Sproull et al., 2019). The relatively high overall prediction values of the current models demonstrate the strength of the algorithm and stability of the SOMAscan assay.

Although key proteomic biomarkers of radiation injury have been established, much remains unknown regarding the complex interaction of injury related pathways following radiation exposure (DiCarlo et al., 2011). The key proteins chosen for model building in this study were selected based on their relative significance within the data set. Yet, most of the selected biomarkers including EDA2R, IGHM, MAPK14, SLC25A18, BAD, CDK2/CCNA2 are not well established biomarkers of radiation exposure. Though changes at the protein expression level of BAD, and at the gene expression level for EDA2R and IGHM following radiation exposure have been reported, these markers are not well characterized as radiation responsive proteins (Chorna et al., 2005; Himburg et al., 2016; Karim et al., 2016). CDK2/CCNA2, SLC25A18, and MAPK14 have not been reported to demonstrate changes in expression directly linked to radiation exposure, though these proteins are indirectly related to radiation induced injury as SLC25A18 is involved in energy metabolism and MAPK14 and CDK2/CCNA1 are involved in DNA damage repair (Liang et al., 2013; Maier et al., 2016; Yoo et al., 2020). E-selectin however, has been reported in multiple studies to demonstrate changes in expression following radiation exposure (Hallahan and Virudachalam, 1997; Liu et al., 2012).

Surprisingly we did not find that established protein biomarkers of radiation exposure such as AMY1, FL, and MCP1, or acute phase reactant proteins such as CRP or SAA were among the top significantly changed proteins within the SOMAscan assay (Ossetrova et al., 2011; Sproull et al., 2017; Balog et al., 2020). One confounder in comparing proteomic expression trends across different technologies is the lack of universal homology in capture or binding molecules. While this confounder has the potential to restrict testing and validation within the same technology, it also provides novel opportunities to discover new radiation responsive biomarkers within specific platforms. Advantages to the SOMAscan platform include its automated high-throughput capacity and its large multiplex approach (>1,300 targets) to proteomic analysis using a small sample volume. This multiplex capacity has also recently been increased to 4,500 targets using the same sample volume which will allow for greater characterization of changes within the mammalian proteome. These current data also establish a data cohort of proteomic expression changes relative to total body radiation exposures in C57BL6 mice within the Somalogic SOMAscan platform. This total body irradiation data can be used as a baseline comparison for future screening of other types of radiation exposures including partial body exposures and organ specific exposures which have more practical value for medical management of radiological or nuclear event exposures. This study presents a novel cohort of protein biomarkers with potential predictive value for radiation exposure.

## Data Availability

The raw data supporting the conclusions of this article will be made available by the authors, without undue reservation, to any qualified researcher.
