# Supplementary figures and images for: Novel Murine Biomarkers of Radiation Exposure Using An Aptamer-Based Proteomic Technology
Source: Front Pharmacol. 2021 Apr 26;12:633131. doi: 10.3389/fphar.2021.633131 (PMC8110031; doi:10.3389/fphar.2021.633131)

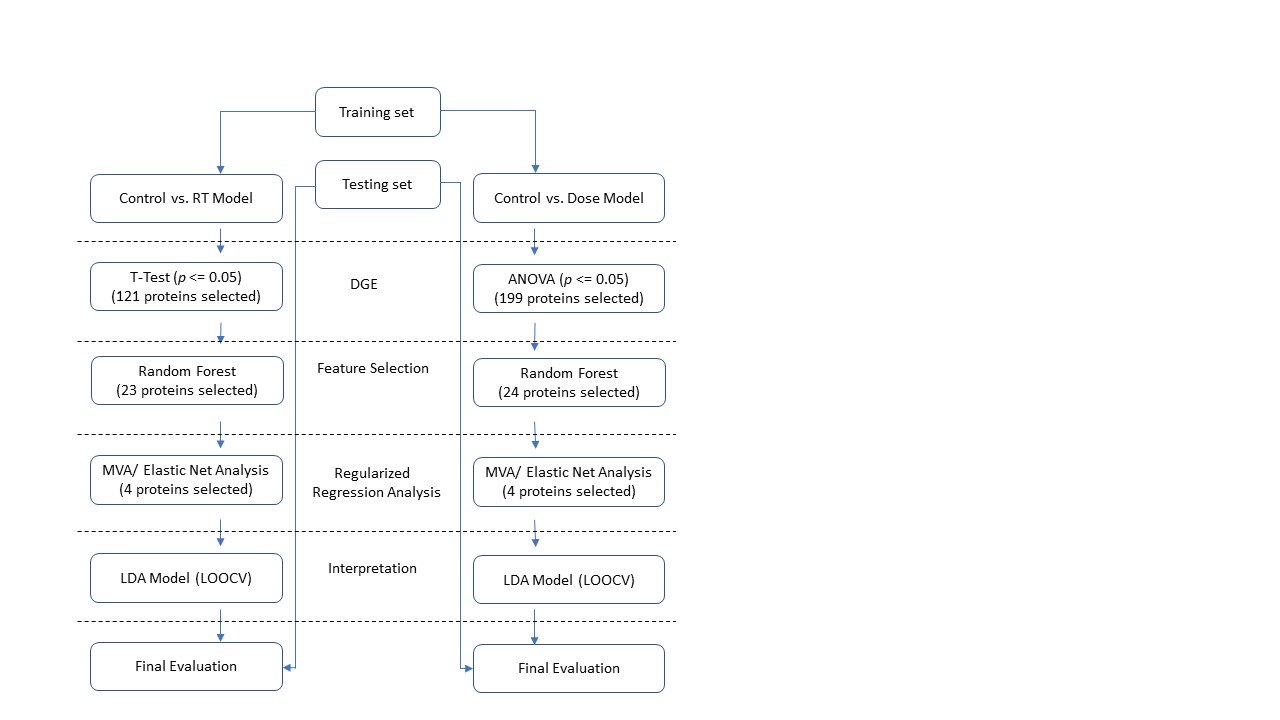

Supplement: Supplementary file 1 [file Image1.JPEG]
